# Multi-Omics Profiling for Health

**DOI:** 10.1016/j.mcpro.2023.100561

**Published:** 2023-04-27

**Authors:** Mohan Babu, Michael Snyder

**Affiliations:** Department of Genetics, Stanford University School of Medicine, Stanford, California, USA

**Keywords:** omics, genomics, transcriptomics, proteomics, metabolomics, lipidomics, exposome, gut microbiome, longitudinal, wearables, diet, electronic health record, data integration, COVID-19, precision health

## Abstract

The world has witnessed a steady rise in both non-infectious and infectious chronic diseases, prompting a cross-disciplinary approach to understand and treating disease. Current medical care focuses on treating people after they become patients rather than preventing illness, leading to high costs in treating chronic and late-stage diseases. Additionally, a “one-size-fits all” approach to health care does not take into account individual differences in genetics, environment, or lifestyle factors, decreasing the number of people benefiting from interventions. Rapid advances in omics technologies and progress in computational capabilities have led to the development of multi-omics deep phenotyping, which profiles the interaction of multiple levels of biology over time and empowers precision health approaches. This review highlights current and emerging multi-omics modalities for precision health and discusses applications in the following areas: genetic variation, cardio-metabolic diseases, cancer, infectious diseases, organ transplantation, pregnancy, and longevity/aging. We will briefly discuss the potential of multi-omics approaches in disentangling host-microbe and host-environmental interactions. We will touch on emerging areas of electronic health record and clinical imaging integration with muti-omics for precision health. Finally, we will briefly discuss the challenges in the clinical implementation of multi-omics and its future prospects.

## Highlights

• Rapid advances in omics and computation are enabling multi-omics deep profiling.
• Multi-omics can capture the complex molecular interplay.
• Multi-omics is revealing insights for personalized health management.
• Multi-omics is poised to revolutionize the future healthcare.

## Graphical Abstract

**Figure:**
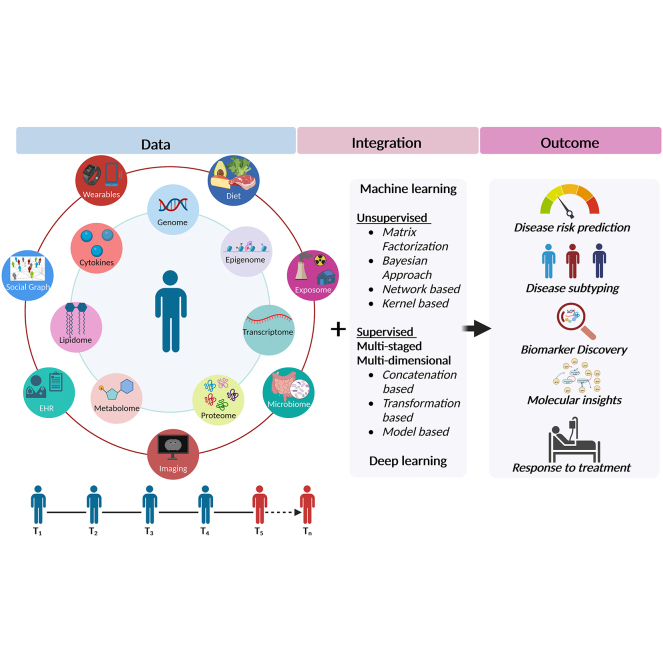

## In Brief

Current medical care focuses on treating people after they become patients rather than to preventing illness, leading to high costs in treating chronic and late-stage diseases. Additionally, a “one-size-fits all” approach to healthcare does not take into account individual differences in genetics, environment, or lifestyle factors, decreasing the number of people benefiting from interventions. Rapid advances in multi-omics enabled deep phenotyping, which profiles the interaction of multiple levels of biology over time, empowers precision health approaches and is poised to transform future healthcare.

The whole is greater than the sum of its parts.

Aristotle

Current medical care primarily focuses on treating patients after the development of illness rather than preventing it, leading to high costs in treating chronic and late-stage diseases. Additionally, common “one-size-fits all” approaches do not take into account individual differences in genetics, environment, or lifestyle factors. This limits the number of people benefiting from known and new interventions. Omics techniques are comprehensive assessments of different classes of biological molecules, such as RNA or metabolites, that have revolutionized modern medicine by advancing our understanding of molecular complexity in health and disease (1). Individual omics approaches, such as genetic sequencing of cancers, are increasingly used in clinical settings and have greatly facilitated disease diagnosis and the identification of biomarkers to track disease or recommend effective treatments (2, 3, 4, 5). However, individual omics data for only one type of biology is largely correlative in nature and cannot capture the complexity of molecular events and their interactions. For example, genome-wide association studies (GWAS) have identified thousands of risk loci for several diseases, yet the causal gene is often not identified, limiting the clinical utility of such findings (6). Combining transcriptomics and proteomics or other omics can provide functional information that cannot be captured by genomics alone, enabling a new understanding of the molecular complexity underlying disease.

Advances in different omics technologies, such as proteomics and metabolomics, and computing capabilities, have recently enabled novel integration of different omics data, called multi-omics, to capture the complex molecular interplay of health and disease by combining the power of individual data types (7). Since complex diseases often develop gradually over time and show incredible heterogeneity between individuals, longitudinal sampling and integrative multi-omics analysis enable deep phenotyping of individuals across the health-to-disease trajectory to unlock precision health approaches for earlier and/or more effective intervention. Precision health aims to predict, prevent, and cure disease more precisely by taking into account each individual’s genetics, environment, and lifestyle factors in contrast to the “one-size-fits all” and reactive approach of traditional medicine. Additionally, by enabling more effective treatment for each patient based on their precise subtyping, this approach could also improve healthcare efficiency and quality. Here, we provide a brief overview of emerging omics technologies and their multi-omics applications in precision health areas including cardio-metabolic diseases, cancer, pregnancy, and longevity. Throughout, we discuss the advantages of multi-omics, where one omics technology can complement the shortcomings of another to provide a holistic view of molecular complexity. Finally, we briefly review emerging omics frontiers, discuss challenges in the clinical implementation of multi-omics, and highlight future prospects.

## Omics (R)evolution

**Fig. 1:**
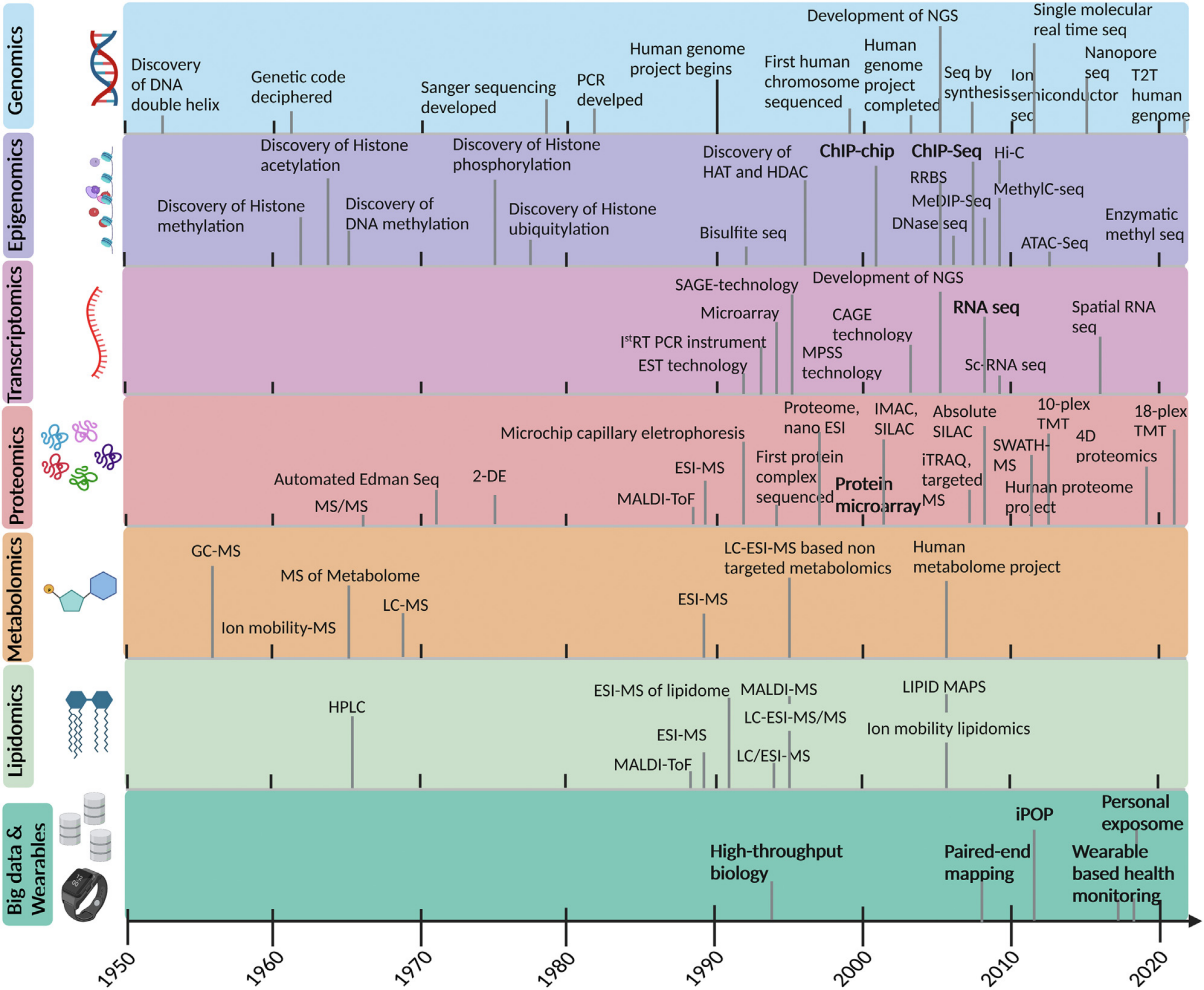
**Timeline of major technological developments and milestones in different Omics analysis.** Technologies developed in the lab are highlighted in bold. 2-DE, 2-Dimensional Electrophoresis; ATAC, Assay for Transposase-Accessible Chromatin; CAGE, Cap Analysis Gene Expression; DNMT1, DNA Methyl Transferase one; ESI, Electrospray Ionization; EST, Expressed Sequence Tags; GC, Gas Chromatography; HAT, Histone Acetyl Transferase; HDAC, Histone Deacetylase; Hi-C, High-throughput Chromosome Conformation Capture; HPLC, High-Performance Liquid Chromatography; IMAC, Immobilized Metal Affinity Chromatography; iTRAQ, Isobaric Tags for Relative and Absolute Quantitation; LC, Liquid Chromatography; MALDI, Matrix-Assisted Laser Desorption/Ionization; MeDIP, Methylated DNA Immunoprecipitation; MPSS, Massively Parallel Signature Sequencing; MS, Mass spectrometry; NGS, Next Generation Sequencing; PCR, Polymerase Chain Reaction; SAGE, Serial Analysis of Gene Expression; Sc-RNA, Single cell RNA; SILAC, Stable Isotope Labeling with Amino acids in Cell culture; T2T, Telomere-To-Telomere.

Modern biology and medicine are being propelled by ongoing advancements in DNA sequencing, mass spectrometry, wearable technologies, and big data computational approaches (Fig. 1). Each of these techniques has been progressing rapidly in the past 20 years:

### Genomics

Genomics is the most mature of all the omics technologies and refers to the study of whole genome sequences and DNA sequence variants therein, including single nucleotide variations, insertion-deletions, structural variations, and copy number alterations. Genomics analysis has seen dramatic progress since the discovery of “Sanger sequencing” of DNA in 1977 (8). With the advent of next-generation sequencing (NGS) technologies in the past couple of decades, genomes can now be analyzed faster, cheaper, and in a high-throughput manner. Genome sequencing costs have plummeted steadily from billions of dollars to sequence the initial human genome in 2000 to just $100 per genome in 2022 (Ultima Genomics). While it took 13 years to sequence the first human genome, patients’ genomes can now be sequenced in as few as 5 h with long-read sequencing techniques, speeding up genetic diagnosis and treatment (9). A prime example of NGS approaches to genomics is high-throughput and massive paired-end mapping, which was used to reveal that genomic structural variation among humans is much larger than initially hypothesized, paving the way for improved understanding of phenotypic variation and genetic disease (10). Genome analyses have already had a major impact on medicine with applications in the diagnosis of diseases, response to treatment, and prognosis (11), such as the identification of treatment interactions and the development of cancer drugs such as erlotinib targeted to specific genetic mutations.

### Epigenomics

Epigenomics refers to the complete cataloging of chemical modifications of DNA and the histones it is wound around. The field of epigenomics began with the discovery of DNA methylation (12) and histone modifications in the 1960s (13) and was accelerated by NGS technologies. Different NGS techniques such as DNA methylation through techniques, including bisulfite sequencing (14), reduced representation bisulfite sequencing (15), methyl-seq (16), methylated DNA immunoprecipitation (17), and enzymatic methyl sequencing (18) have enabled precise mapping of genome-wide methylation patterns and other epigenetic markers that affect gene regulation. The histones around which DNA is wound are composed of dimers made up of four basic proteins, H2A, H2B, H3, and H4, which undergo myriad post-translational modification, including acetylation, methylation, phosphorylation, and sumoylation, that affect how certain genes are turned on or off. Early technologies to analyze histone modifications used immunoprecipitation with antibodies against specific histone modification sites on DNA but were limited by expense and throughput. The development of ChIP-chip, where genomic DNA sites enriched in specific modifications were identified by DNA hybridization to a microarray (ChIP-chip) (19, 20, 21, 22, 23), improved epigenomics studies. This approach, however, was noisy and expensive to apply genome-wide. The advent of NGS further enabled high-resolution genome-wide mapping (ChIP-seq) of chromatin modifications and the location of the bound regions (24, 25, 26). Epigenomics, being an integrator of genome and environment, has also found wider application in disease diagnosis, prognosis, and therapy (27).

### Transcriptomics

Transcriptomics measures the complete set of RNA transcripts and their quantity in a cell or a population of cells as a read-out of cell state (28). Progress in transcriptomics has paralleled rapid developments in NGS and analysis technologies (28). Initial transcriptomics approaches used both hybridization-based (Microarray) and sequencing-based approaches for the quantification of transcripts. However, microarray technologies could only detect genes and exons previously incorporated into the array and could not detect novel transcripts. Additionally, low-expressed genes were not detectable (sensitivity), and microarrays failed to differentiate between genes with sequence homology (specificity) (29, 30). In contrast, sequence-based approaches, such as serial analysis of gene expression or massively parallel signature sequencing, were limited in their ability to detect all transcript isoforms and were expensive (31, 32). Several studies in 2008 reported high-throughput sequencing of the whole transcriptome, known as RNA sequencing (RNA-Seq), which revealed new parts of the genome that are transcribed while also enabling more accurate RNA quantitation, detection of transcripts with low expression, and identification of new genes, exons, and transcript isoforms at the same time (33, 34). Continued progress in transcriptomics has also revolutionized modern medicine with applications in disease diagnosis and prognosis, enabling the definition of how different genes interact in unique cell types over time (35).

### Proteomics

Proteomics, the quantification of all protein identity and abundance in a sample, has similarly seen major advances in technologies and instrumentation, enabling faster, more efficient, sensitive, and accurate detection of proteins (36). Mass spectrometry (MS)-based proteomics began with the development of soft ionization techniques such as electrospray ionization (ESI) (37) and matrix-assisted laser desorption ionization (MALDI) (38) in volatilizing and ionizing proteins and peptides in the 1990s but was limited in how many proteins could be identified. The first high-throughput analysis of proteins was achieved using protein array methodologies based on prefabricated chips with specified protein detection. Despite being sensitive, this approach could not capture the entire proteome (39). From this followed multidimensional protein identification technology, which used two-dimensional liquid chromatography to separate proteins before tandem-MS analysis (40). After this followed shotgun proteomics with better sensitivity, dynamic range, molecular weight, and hydrophobicity (41). The early 21st century has witnessed significant improvements in both liquid chromatography (LC) and MS parameters, especially the higher scanning frequency and mass accuracy, enabling an era of LC-MS/MS-based “next generation proteomics”. More recent labeling strategies, such as tandem mass tag (42) and isobaric tagging for relative and absolute quantitation (43), offer improved multiplexing and sensitivity to significantly reduce LC-MS analysis times and increase throughput. With rapid, robust, and high-throughput analysis, including options for multiplexing large numbers of samples, the costs of proteome analysis per sample have dropped from $3250 in 2006 to just $375 in 2021 (44). MS-based proteomics has already shown promise by successfully revealing complex and predictive biomarker signatures leading to improved clinical decision-making as well as enabling the prediction of patient trajectories *via* machine learning (45). Furthermore, recent advances in data-independent acquisition methods such as the sequential windowed acquisition of all theoretical fragment ion spectra-MS are expected to transform clinical diagnostics and prognosis through scalable and affordable proteomics (46, 47).

### Metabolomics

Metabolomics refers to the study of small molecules in the body <1500 Da in mass and has similarly seen a dramatic improvement in technologies and instrumentation in the past several decades. Major metabolomics approaches include targeted metabolomics, untargeted metabolomics, fluxomics, and metabolite imaging. Targeted metabolomics aims to identify and quantify a small subset of metabolites (50–500) and is ideal for biomarker detection. Untargeted metabolomics attempts to characterize all possible number of metabolites (>10,000). Fluxomics is a branch of targeted metabolomics that monitors the movement of isotopic labels through metabolic intermediates and measures metabolite reaction rates. Metabolite imaging is an emerging field of metabolomics that involves the detection and visualization of metabolites in tissues (48). Being the substrate on which genetics, environment, microbiota, and exposome interact, metabolomics studies have propelled biomedical research with applications in biomarker discovery, disease diagnosis, and prognosis (4). Lipidomics in particular has seen significant progress with MS-based technological advances. Analysis of intact cellular lipids was greatly accelerated with the advances in ionization technologies, which were in large part fueled by the development of ESI and MALDI in the late 1980s (37, 38). Advances in MS methods have improved both resolution and mass accuracy while multiplexing has enabled exponential growth in lipidomics throughput and utility since the 1990s, offering exciting new possibilities to understand health and disease (49).

### Wearables

Wearable devices (wearables) refer to any miniaturized electronic device with sensors that can be donned on the body or integrated into clothing or other body-worn accessories (50). Wearable technologies are revolutionizing biomedicine through mobile and digital health by enabling continuous, longitudinal monitoring of vital physiological parameters including heart rate, sleep, pulse oximetry, blood pressure, steps, and temperature (51). Along with multi-omics, wearable data can track transitions from health and disease at an exquisite resolution and is considered an important tool for precision health (50, 51). Recent studies have demonstrated the potential of wearables in detecting inflammation, predicting cardiometabolic health, and passively predicting atrial fibrillation (52, 53, 54, 55, 56). In fact, measurements from consumer smart watches could reliably predict clinical measurements of inflammation, infection, and even insulin sensitivity status. Continuous glucose monitoring could longitudinally track glucose dynamics and uncover highly personal glucotypes to provide nutritional guidance (57). More recently, smart watch-based physiological monitoring was shown to successfully detect symptomatic and pre-symptomatic COVID-19 infections (58, 59, 60). This shows promise in expanding the use of wearables in clinical applications for detecting both acute health events and for monitoring and managing chronic diseases.

## Applications in Precision Health

**Fig. 2:**
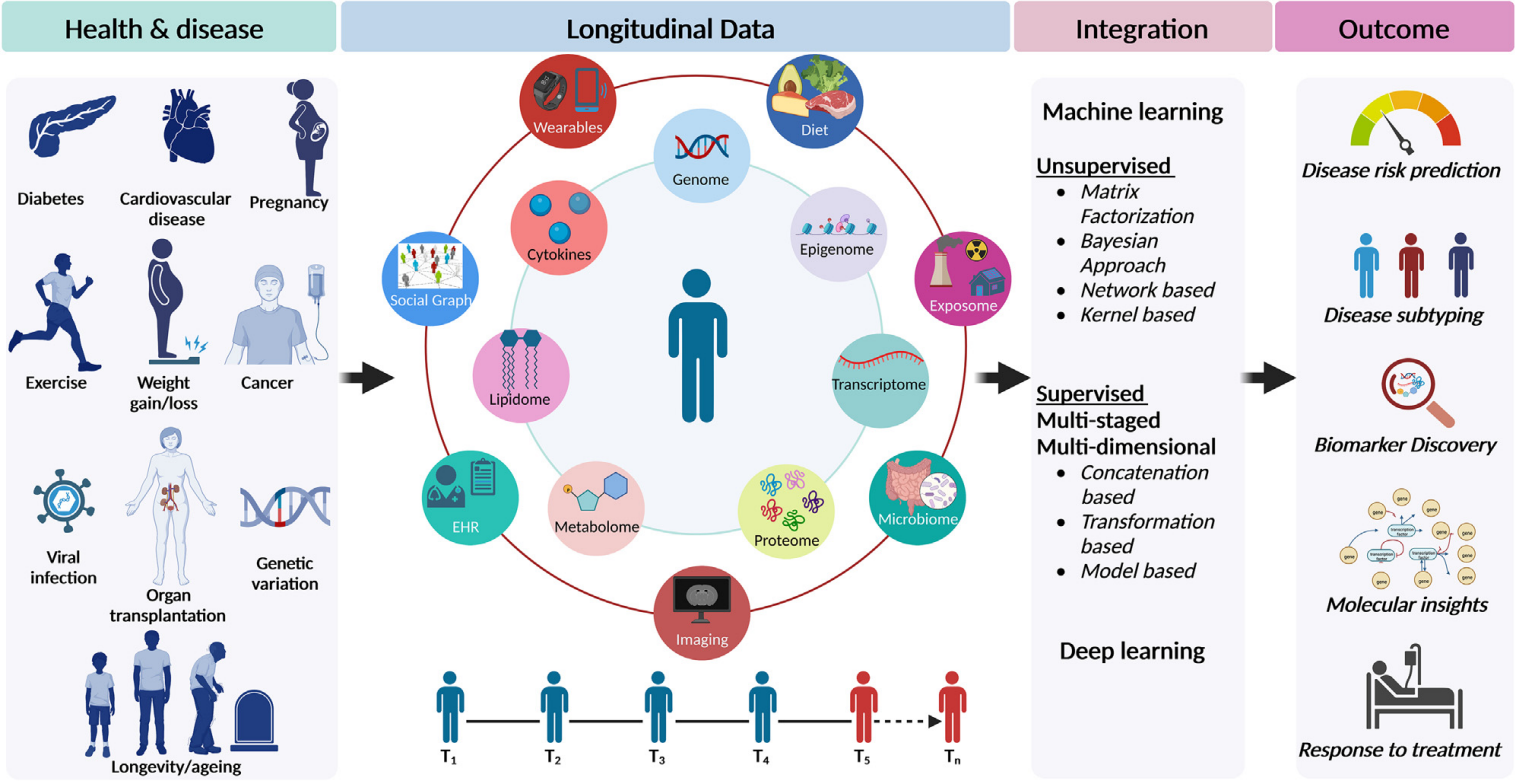
**Longitudinal multi-omics and wearable data enabled deep phenotyping for precision health.** Omics and non-omics data across times (T_1_-T_n_) could be integrated using machine learning and deep learning approaches to predict disease risk, subtyping, biomarker discovery, molecular insights, and response to treatment among others. Figure was created using Biorender (https://biorender.com/).

Broadly, multi-omics integrative approaches have been critical in (1) predicting disease risk, (2) disease subtyping (*e.g.*, glucotypes, ageotypes) and classification, (3) biomarker discovery, (4) deriving biological insights, and (5) stratifying patients for therapy (*e.g.*, mild, moderate, and severe COVID-19) among others (Fig. 2). Multi-omics integrative approaches have enabled deep phenotyping of individuals in health and disease, leading to many clinically actionable discoveries (61, 62, 63). A prime example of this is a longitudinal integrative personal omics profiling (iPOP) study performed on a 54-year-old individual at 20 time points over a 14-month period. This study characterized the transition from a healthy to an insulin-resistant state following a viral infection and uncovered extensive, dynamic changes in diverse molecular components and biological pathways during this transition, prompting lifestyle changes in the individual and quantifying their impact (64). Further biological pathway expression analysis integrating metabolomics and proteomics data in more patients was found to predict and monitor disease (65). Since then, the iPOP study has been expanded to >116 individuals for health discoveries and molecular understanding of response to perturbations including weight gain/loss, exercise, and vaccination.

In a similar vein, the Pioneer 100 Wellness Project (P100) initiated by the Institute of Systems Biology studied 108 individuals over the course of 9 months to create a personal, dense, and dynamic data cloud for each individual. Further analysis of inter-omics correlations led to the identification of putative biomarkers for cardiometabolic disease (55). The Pioneer 100 study has been expanded to 100,000 participants in 100K Wellness Project examining blood, saliva, and stool as well as other physiological and psychological measurements to capture the initiation and progression of many common diseases. A longitudinal integrated multi-omics, physiological and behavioral analysis performed on a pair of monozygotic (identical) twin astronauts (One twin on board International space station and the other on Earth) for the first time revealed the impact of long-duration space flight on human body. The molecular insights revealed pathways and mechanisms that are vulnerable to spaceflight and could serve as a guide for targeted countermeasures/monitoring during future missions (66). Longitudinal saliva multi-omics was used to monitor immune response to vaccination is emerging as a non-invasive diagnostic approach (67). Together these initial studies showcase the immense potential of multi-omics approaches for assessing health status, discovering clinically actionable insights into illness, and guiding personalized medical treatment to ultimately provide better health management.

## Genetic Variation to Disease

The completion of the Human Genome Project marked the beginning of a new era in biomedical research (68). This was followed by large-scale GWAS for identifying thousands of genetic variations associated with diseases or complex traits. However, the functional relationship of these variations to patient phenotype or the translation of GWAS results to clinical applications has been lacking. Since most GWAS loci fall within non-coding regions, assigning functions to these variants has been challenging (69, 70, 71). In this regard, multi-omics integration of whole-genome sequencing (WGS) or whole-exome sequencing (WES) data with transcriptome information has been critical in identifying genes and pathways that may have a role in a particular disease. Importantly, proteomics has revealed the effect of genetic variants in conditions otherwise undetectable by RNA analysis (72). For example, through integrative analysis of ribosome profiling, RNA sequencing, and MS of lymphoblastoid cell lines from 95 ethnically diverse individuals, we discovered distinct mechanisms of gene expression variation among humans and found that genetic variants can cause changes in protein levels through effects on translation (73).

While WGS and WES can characterize ∼10,000 variants per genome, computational algorithms for accurately predicting and prioritizing functional pathogenic variants have been challenging. Towards this end, Mohammadi *et al.* (74) recently developed a new method, ANEVA-DOT test, to compare the expression activity of maternal and paternal alleles to identify heterozygous DNA variants with a strong effect on gene expression in rare genetic diseases and other complex conditions. Multi-omics approaches can be utilized for the construction of gene-regulatory networks to prioritize disease-risk genes and for the prediction of drug efficacy (75). Visscher and Yang have developed a method called omics-data-based complex trait analysis to identify associations between omics data, such as DNA methylation, and complex traits while also accounting for confounding factors (76). Marioni *et al.* carried out both GWAS and epigenome-wide association studies on 92 plasma proteins with known neurological links from 750 healthy older adults and identified both genetic and epigenetic factors associated with the protein biomarkers (77). Similarly, a multi-omics approach integrating functional genomics with GWAS summary statistics identified 650 amyotrophic lateral sclerosis-associated genes that represent a fivefold increase in recovered heritability, extensive conservation, and transcriptome network changes associated with disease development. Rare variant analyses have demonstrated the functional significance of candidate genes in healthy and diseased motor neurons and brain tissues. These studies demonstrate the power of multi-omics in dissecting the genetic basis of complex diseases that were not possible with single omics approaches, opening new avenues for precision interventions (78).

## Cardio-Metabolic Disease

Complex diseases including cardiovascular, metabolic disorders, and cancer evolve over time and show incredible heterogeneity among individuals. Thus, longitudinal multi-omics integrative analysis can identify temporal molecular shifts indicative of physiological transitions between healthy to disease states (Fig. 2). A prime example of this is a longitudinal multi-omics study of weight perturbation that demonstrated activation of strong inflammatory and hypertrophic signatures in the blood associated with weight gain. Although weight loss reversed some changes, several signatures persisted, indicative of long-term physiological changes due to weight gain. Additionally, omics signatures revealed an association with insulin resistance that could serve as a novel diagnostic. Interestingly, specific biomolecules were highly individualized and stable in response to perturbations, potentially representing “personalized biomarkers” (79). In a similar vein, the prediabetes to clinical type 2 diabetes mellitus (T2DM) transition was captured by performing multi-omics on prediabetic individuals for over 4 years. This rich longitudinal data set revealed many insights into precision health: first, healthy profiles were distinct among individuals while displaying diverse patterns of intra-and/or inter-personal variability. Second, extensive host and microbial changes were found during respiratory viral infections while immunization was found to trigger potentially protective responses that are distinct from responses to respiratory viral infections. Moreover, during respiratory viral infections, insulin-resistant (IR) participants’ immune signatures responded differently than insulin-sensitive (IS) participants. Third, global co-association analyses among the thousands of profiled molecules revealed specific host-microbe interactions that differed between IR and IS individuals. Lastly, this study identified early personal molecular signatures in one individual that preceded the onset of T2DM, including the inflammation markers interleukin-1 receptor agonist and high-sensitivity C-reactive protein paired with dysregulated xenobiotic-induced immune signaling. Overall, this study revealed insights into myriad pathways and responses that differ between glucose-dysregulated and healthy individuals during health and disease (80).

A recent study used deep longitudinal multi-omics profiling including emerging technologies like immunome, microbiome, and wearable monitoring of a cohort enriched for risk factors for T2DM for up to 8 years and discovered more than 67 clinically actionable health discoveries while also identifying multiple molecular pathways associated with metabolic, cardiovascular, and oncologic pathophysiology. Additionally, omics measurements could reliably predict insulin resistance, illustrating their potential to replace burdensome clinical tests (81). Similarly, multi-omics has also been applied to understand the molecular basis of hypertrophic cardiomyopathy (HCM). Comprehensive molecular analysis using transcriptome, metabolome, and lipidome profiling of myocardial samples from HCM and normal controls revealed perturbed metabolic signaling and mitochondrial dysfunction as common pathogenic mechanisms underlying HCM, highlighting potential new drug targets for attenuation of HCM (82).

## Cancer

Cancer etiology is multifactorial and highly heterogenous in nature, requiring multi-modal approaches to dissect its underlying mechanisms and develop new therapies. In this direction, Liu *et al.* (83) utilized multi-omics analysis of genomic CNVs, DNA methylation, and gene expression in 256 hepatocellular carcinoma samples and identified five subgroups with distinct molecular signatures and a distinct survival rate. Kamoun *et al.* (84) performed multi-omics integrative analysis on oligodendroglial tumors to identify three subgroups of 1p/19q co-deleted gliomas. Single omics measurements have been used in melanoma prognosis prediction. Unfortunately, these approaches cannot comprehensively describe the biological processes underlying prognosis and the prognostic models developed were less accurate for clinical implementation. To this end, Jiang *et al.* (85) performed an integrative analysis of clinical variables, genomic CNVs, DNA methylation, and gene expression data from The Cancer Genome Atlas and found that integrated analysis led to models with improved prediction, with a mean C-statistic of 0.724. Although chromatin alterations are reported in several cancers, their relevance for cancer gene expression phenotypes remains unclear. Recently, multi-omics profiling of chromatin accessibility, RNA, and protein abundance of human thyroid cancer primary tumors, metastases, and patient-match normal tissue identified gene body enhancers predictive of correlated RNA and protein expression. This study demonstrates the utility of multi-omics in identifying potential targets and better understanding cancer treatments (86). In a similar vein, Zhang *et al.* (87) performed genome sequencing and proteomics analysis on high-grade ovarian serous carcinomas to unravel the influence of different gene copy-number variations on the proteome, post-translational modification levels, and clinical outcomes, providing a mechanistic link between copy-number variations and potential progression events in ovarian cancer. These studies demonstrate the power of multi-omics in understanding mechanisms of cancer and subtyping for precision therapies. Recent reviews provide an excellent and in-depth overview of multi-omics applications in cancer (88, 89, 90).

## Infectious Diseases

Multi-omics have enabled deep characterization of antibody responses to infections and vaccines. Bulk and single-cell multi-omics were instrumental in dissecting cellular responses to SARS-CoV-2 viral infection and subsequent vaccine responses during the COVID pandemic. While most investigations involved focused on analyzing one information layer at a time (*e.g.*, fluorescence-activated cell sorting) to understand the dynamics of circulating immune cells in COVID-19, Bernardes *et al.* performed longitudinal multi-omics on peripheral blood mononuclear cells (PBMCs) from patients with COVID-19 infection throughout the disease course for a comprehensive understanding of longitudinal cellular features. Interferon-activated circulating megakaryocytes and increased erythropoiesis coincided with critical illness while megakaryocytes- and erythroid-cell-derived co-expression modules were predictive of fatal disease outcomes. This multi-omics approach demonstrated the broad cellular effects of SARS-CoV-2 infection at the epigenetic and transcriptional level beyond just phenotypic analysis of immune cells providing insights to develop biomarkers and precision treatments for patients with COVID-19 (91). A recent study using an integrated single-cell multi-omics profiling of human lungs discovered and validated over 1000 risk genes underlying severe COVID-19 across 19 cell types. Genetic risk for severe COVID-19, covering both common and rare variants, was particularly enriched in natural killer cells. Further, RefMap, a machine learning algorithm, enabled sensitive prediction of severe disease in non-elderly patients based on GWAS and single-cell omics. Individualized predictions were accurate independent of age and sex and were consistent across multiple populations and cohorts. When combined with machine learning, this single-cell multi-omics approach provided novel insights into the molecular mechanisms of severe disease, leading to new therapeutic targets and sensitive detection of at-risk individuals (92). Similarly, Sacco *et al.* applied longitudinal multi-omics (analysis of soluble biomarkers, proteomics, single-cell gene expression, and immune repertoire analysis) to identify immunopathological signatures between pediatric COVID-19 and multisystem inflammatory syndrome in children (MIS-C). Pediatric COVID-19 was characterized by robust type I interferon (IFN) responses, whereas increased levels of circulating spike protein, matrisome activation, and prominent type II IFN-dependent or NF-kB-dependent signatures were detected in MIS-C. This approach thus better defines the pathophysiology of these disorders and helps design precision therapies (93).

Multi-omics has also been used to characterize the molecular shifts between mild and moderate COVID-19. By characterizing circulating immune cell classes and plasma multi-omics profiles form two longitudinal blood draws, Su *et al.* demonstrated elevated inflammatory signaling accompanied by loss of specific classes of metabolites and metabolic processes during a shift from mild to moderate COVID-19 disease. This integrated approach revealed that moderate disease may provide the most effective setting for therapeutic intervention (94). Multi-omics approaches are also accelerating vaccine development by helping construct global maps of the complex immune responses that occur during vaccination to identify cellular and molecular correlates of vaccine efficacy (For a comprehensive overview of multi-omics approaches for precision medicine in infectious diseases, See Refs (95, 96)). MS-based proteomics, with its fast turn-around and high throughput, has been instrumental in revealing classifiers of COVID-19 infection. Recently, Messner *et al.* developed a low-cost platform (less than 10€ for consumables per sample) for ultra-high-throughput serum and plasma proteomics. In a cohort-based epidemiological study, the platform could identify 27 potential markers that revealed the severity grade of COVID-19. The platform demonstrates the power of MS-based large-scale proteomics in clinical decision support in situations needing rapid responses such as the COVID-19 pandemic (97).

## Organ Transplantation

Organ transplantation remains the ultimate treatment option for patients with end-stage disease with organ failure, yet mortality rates are high due to frequent rejection. This is due to a limited understanding of complex post-transplant immune adaptation mechanisms. A better understanding of donor–recipient matching and longitudinal multi-omics tracking after transplant is needed to improve rejection rates and design personalized therapies. Towards this end, Watzenboeck *et al.* combined profiling of the alveolar microbiome, cellular composition, metabolome, and lipidome in bronchoalveolar lavage samples from organ recipients and donors to identify recipient-specific and environmental factors that shape the long-term lung microbiome. The abundance of certain bacterial strains correlated with underlying lung diseases even after transplantation. By applying machine learning models to this data, they could accurately predict changes in forced expiratory volume during the first second (FEV_1_, a major characteristic of lung allograft dysfunction) from multi-omics data, whereby lung microbiome composition showed a high predictive power (98).

Wigger *et al.* conducted a comprehensive multi-omics analysis of pancreatic islets obtained from metabolically profiled pancreatectomized living human donors stratified along the glycemic continuum (from normoglycemia to T2DM) and found remarkable heterogeneity in the transcriptomic and proteomic profiles in patients with diabetes compared to non-diabetic controls. Differential regulation of islet gene expression is already observed in prediabetic individuals with impaired glucose tolerance, suggesting a progressive, but disharmonic, remodeling of mature beta cells and thus challenging the current model of a linear trajectory toward precursor or transdifferentiation stages in T2DM development. Furthermore, through the integration of islet transcriptomics with preoperative plasma lipidomics, this study also defined the relative importance of gene coexpression modules and lipids that are positively or negatively associated with HbA1c levels, pointing to potential prognostic biomarkers. This approach helps define subtypes of T2DM, and biomarkers thereof, thus enabling precision approaches for the treatment of T2DM (99).

## Pregnancy

Multi-omics approaches have also been instrumental in unraveling biological signatures and transitions predictive of pregnancy-related complications, including pre-term birth (PTB) and preeclampsia. Multi-omics modeling integrating transcriptomic, immunological, microbiomic, metabolomic, and proteomic measurements during the course of full-term pregnancy was used to measure the ability of each dataset to predict gestational age. Among the individual dataset, plasma proteomics had the strongest predictive power. Additionally, combining all datasets increased the predictive power and revealed novel interactions among different biological modalities (100). Longitudinal multi-omics (metabolome, proteome, and immunome) profiling captured a distinct molecular shift from pregnancy maintenance to pre-labor biology occurring 2 to 4 weeks before delivery. A surge in steroid hormone metabolites and interleukin-1 receptor type 4 preceded labor onset and coincided with a switch from immune activation to the regulation of inflammatory responses. This approach could help in developing blood-based methods predicting the day of labor, anchored in mechanisms shared in pre-term and full-term pregnancies (101). Multi-omics analysis combined with machine learning modeling was also used to identify early biological measurements associated with pre-term birth in five biorepository cohorts in low- and middle-income countries (102). These studies reveal the power of combining multi-omics and machine learning for developing valuable predictive tests and intervention candidates for preventing PTB.

## Longevity/Aging

Longitudinal multi-omics profiling was also used to reveal myriad molecular changes during aging, identifying both known and new markers, as well as distinct molecular patterns of aging in insulin-resistant as compared to insulin-sensitive individuals. Molecular pathways that changed over time in each individual suggested different aging patterns (ageotype) that may ultimately be useful in monitoring and intervening in the aging process (45). In a similar vein, Nie *et al*. utilized multi-omics data, including clinical tests, immune repertoire, targeted metabolomics, gut microbiome, physical fitness tests, and facial skin examination to estimate the biological ages of different organs to identify diversity in aging. This study revealed different aging patterns across the study population, suggesting precision interventions may be necessary to decrease the impact of aging (103). In another study, multi-omics profiling was used to understand variability in reprogramming old or young fibroblasts to induced pluripotent stem cells (iPSC) akin to ageotypes. This approach revealed that fibroblast cultures from older mice contained “activated fibroblasts” that secrete inflammatory cytokines and that the proportion of activated fibroblasts in each cell culture correlated with the reprogramming efficiency. This could help in developing personalized strategies to improve iPSC cell generation and wound healing in elderly individuals (104). These studies highlight the promise of multi-omics integrative approaches in developing personalized aging interventions.

## Emerging Frontiers in Precision Health

### Host–Microbiome Interactions

The microbiome, often considered our second genome, shapes health and plays a crucial role in a plethora of diseases. Characterization of diverse microbes in healthy individuals found extensive variation in both body site habitat and between different individuals, giving rise to the concept of a “personal microbiome” (105). Microbial interactions with their human hosts change across health and disease and thus serve as a modifiable factor to manage health (106). A recent host-microbial multi-omics study demonstrated taxonomic and functional differences between insulin-resistant and insulin-sensitive individuals in various measurements, both at baseline and in response to stresses such as weight loss and respiratory viral infections (80). Along similar lines, Heintz-Buschart *et al.* (107) performed microbial multi-omics on four families with type 1 diabetes and observed intra- and inter-individual variation demonstrating a pronounced effect of family membership on the structural and functional composition of the gut microbiome. Lloyd-Price *et al.* performed an integrated host-microbial multi-omics longitudinal profiling study that provided a comprehensive view of functional dysbiosis in the gut microbiome during inflammatory bowel syndrome activity. They demonstrated that a characteristic increase in facultative anaerobes at the expense of obligate anaerobes, as well as molecular disruptions in microbial transcription, metabolite pools, and levels of antibodies in host serum, was correlated with the development of bowel inflammation. Periods of disease activity were also marked by increases in temporal variability in the microbiome with characteristic taxonomic, functional, and biochemical shifts. Finally, the integrative analysis identified microbial, biochemical, and host factors central to this dysregulation (108). Similarly, through longitudinal sampling and integrative host–microbial multi-omics, a recent study identified inflammatory bowel syndrome subtype-specific and symptom-related variations in microbial composition and function. Furthermore, purine metabolism was identified as a key host–microbial metabolic pathway as a therapeutic target for inflammatory bowel syndrome (109). Thasis *et al.* utilized host methylome, transcriptome, metabolome, and gut microbial metagenome and imaging data to quantify the global reprogramming of host biology by microbiota. They showed a tight link between the host and microbial circadian activities and further found that disruption of microbial rhythmicity abrogates normal host oscillations in the intestine and liver, influencing host diurnal fluctuations (110). Apart from host-microbial omics analysis, extra-cellular vesicles (EVs) secreted by host and microbial cells are emerging as critical players in cell-to-cell communication under various conditions. EVs are lipid bilayerd structures containing transmembrane proteins, cytosolic proteins membrane-associated proteins, and nucleic acids. Upon release by cells, EVs can interact with adjacent or distant cells and modulate their function through signaling *via* surface contact or by transferring cargo (111). A comprehensive omics profiling of EVs thus could serve as biomarkers for a range of clinical conditions including COVID-19 (112), cancers (113), and so on. These studies demonstrate the power of integrated longitudinal host–microbial multi-omics analyses in revealing the complex interactions that shape host physiology and may be amenable to precision interventions for preventing diseases.

### Host–Environmental Interactions

Genetic loci identified from GWAS have been able to explain only a small proportion of complex disease heritability/etiology, leading to “missing heritability” concept (114). Non-genetic factors including lifestyle, diet, and environmental exposures (exposome) are suggested to explain the “missing heritability” of complex diseases (115). Lifestyle factors, especially physical exercise, favorably impact overall health and protect against complex diseases including obesity, diabetes, and other cardiometabolic diseases (116, 117, 118, 119). However, the molecular mechanisms underpinning exercise-induced benefits have not been clearly defined. Along this direction, a longitudinal multi-omics profiling study of plasma and PBMCs from 36 well-characterized volunteers, before and after a controlled bout of symptom-limited exercise, detected distinct molecular changes and an orchestrated choreography of biological processes involving energy metabolism, oxidative stress, inflammation, tissue repair, and growth factor response as well as regulatory pathways governing magnitude and duration of those responses. Interestingly, these processes were dampened, and some were even reversed, in insulin-resistant participants. Machine learning models based on multi-omics data from this study were able to predict potential blood-based biomarkers of peak oxygen consumption during exercise (120). Recently, Li *et al.* (121) discovered an exercise-induced metabolite, *N*-lactoyl-phenylalanine (Lac-Phe), as a suppressor of feeding and obesity in mouse, humans, and racehorse models of exercise and provided new insights into molecular responses to physical activity. To tap the immense potential of multi-omics, datasets with larger samples and more tissue- and disease-specific repositories are essential. In this direction, a larger consortium involving pre-clinical and clinical studies is also examining systemic response to acute and chronic exercise using multi-tissue multi-omics. This will serve as a public database to enhance our understanding of the health benefits of exercise and could provide insights into how exercise mitigates disease (122).

Diet, another lifestyle factor, has a profound impact on host physiology and exerts a “personalized effect” (123). Integrative multi-omics, wearable data, and machine learning approaches are revealing molecular insights into individuals’ responses to diet, enabling improved precision nutrition approaches. For example, Zeevi *et al.* (124) created a machine learning algorithm that included blood parameters, dietary habits, anthropometrics, physical activity, and gut microbiome data that could accurately predict personalized postprandial glycemic response to real-life meals. Recently, Berry *et al.* assessed postprandial metabolic responses in ∼1002 twins and unrelated healthy adults and found a large interindividual variability in blood triglycerides, glucose, and insulin. Machine learning models implementing meal composition, habitual diet, meal context, anthropometry, genetics, microbiome, clinical, and biochemical parameters could accurately predict postprandial triglyceride and glucose responses (125). A recent study used continuous glucose monitoring (CGM) to longitudinally track glucose dynamics in response to standardized meals and uncovered highly personal glucotypes unique to participants (57). In a similar vein, personalized responses to dietary fiber (arabinoxylan & inulin) supplementation were also discovered using host multi-omics, microbiome, and clinical parameters (126). These studies show the utility of integrative multi-omics, microbiome, and machine learning approaches to dissect individual responses to diet.

The exposome is another non-genetic modifier of health that includes both biological (*e.g.*, pollen, viral particles) and chemical components (*e.g.*, pollutants, disinfectants, and insecticides). This diverse repertoire of components can exert distinct biological responses through methylation, gene expression changes, microbial shifts, and inflammatory cytokine secretion (127). The external exposome can influence internal omics responses, including metabolomics, linking functional environmental changes to chronic disease (128). However, the impact of diverse environmental exposures on individuals’ health is not clearly understood and thus needs large-scale efforts comparable to human genome sequencing (129). To this end, a recent study longitudinally profiled the personal exposome of 15 adult individuals for up to 890 days using a portable exposometer. Combined with deep sequencing and mass spectrometry profiling, over 2500 microbial species and 2796 putative chemical features were identified in these collected personal airborne exposures and showed highly dynamic changes in exposome composition in response to varying environments and lifestyles (130). Similarly, a recent study from Human Early Life Exposure (HELIX) project investigated the biological effects of early life exposure in a multicenter cohort of 1301 mother–child pairs and associated individual exposomes consisting of 100 chemical, physical, and lifestyle exposures assessed in pregnancy and childhood followed by multi-omics profiling in childhood. They identified 1170 associations, 249 in pregnancy and 921 in childhood, which revealed potential biological responses and sources of exposure. The methylome best captured the persistent influence of pregnancy exposures, including maternal smoking, while childhood exposures were associated with features from all omics layers, revealing novel signatures for indoor air quality, essential trace elements, endocrine disruptors, and weather conditions (131). To better understand how the exposome shapes an individual’s phenotype, a recent study used deep longitudinal personal exposome and internal multi-omics profiling and annotated thousands of chemical and biological components in the personal exposome cloud, finding a significant correlation with thousands of internal biomolecules which were cross-validated using corresponding clinical data. These results showed that agrochemicals and fungi dominated the highly diverse and dynamic personal exposome, while the biomolecules and pathways related to the individual’s immune system, kidney, and liver were most highly associated with their personal external exposome. This data-driven longitudinal monitoring study showed the depth of dynamic interactions between the personal exposomes and internal multi-omics, underlining the need for further study and tool development (132).

## Wearable and Electronic Health Record Data Integration

Electronic health records (EHR) can complement multi-omics and wearables with longitudinal clinical data, including diagnostic codes, procedure codes, lab results, physical measurements, clinical notes, and medical images. In this direction, a recent study leveraged a machine learning framework to integrate genomes, EHR data, and lifestyle factors to accurately predict the occurrence of abdominal aortic aneurysm (133). Despite its clinical utility, EHR data are usually sparse with records from discrete clinical visits. Thus, wearable-based continuous physiological monitoring and integration with other multi-omics data within EHR will be critical to speed up clinical decisions and potentially reduce medical costs. For instance, initiatives by the NIH such as the “All of US” project are building health databases collecting EHR, questionnaires, physical measurements, digital health technologies, and the collection and analysis of biospecimens of a million diverse individuals that will characterize the intersection of biology, lifestyle, and environment in health (134).

## Large-Scale Efforts Advancing Multi-Omics Enabled precision health

Multi-omics–enabled precision health is propelled by continued technical advances in human genomics, proteomics, lipidomics, and metabolomics. For instance, long-read sequencing has enabled the completion of the human genome with telomere-to-telomere sequencing (T2T Consortium) and will provide a gold standard reference for mapping genetic variation to the genome and detecting pathogenic variants (135). Similarly, the human proteome project launched by The Human Proteome Organization (HUPO) in 2010 has made enormous progress in enhancing accurate annotation of genome-encoded proteins and reached a 90.4% complete high-stringency human proteome blueprint in 2021 (136). As a part of HUPO, we quantified the relative protein levels from over 12,000 genes across 32 normal human tissues, identified tissue-specific and tissue-enriched proteins, and compared them to transcriptome data. Discordance of RNA and protein levels revealed potential sites of protein synthesis and action of secreted proteins. Most importantly, our study demonstrated that protein tissue-enrichment information can explain phenotypes of genetic diseases that cannot be obtained by transcript information alone. Furthermore, we demonstrated how understanding protein level patterns can provide insights into gene regulation, the secretome, metabolism, and human diseases (137). Similarly, the human metabolome database (HMDB) created by the Human Metabolome Project has curated detailed information on small molecule metabolites found in the human body, serving as an up-to-date reference database for metabolomics studies (138).

Multi-omics repositories are providing a rich data resource to understand health and disease at the population level (Table 1). For example, two large-scale epigenetics studies, the Encyclopedia of DNA Elements (ENCODE) project and Roadmap Epigenomics, have mapped regions of transcription, transcription factor association, histone modification, DNA methylation, and chromatin structure to delineate all functional elements encoded in the human genome (139), and develop (140) critical reference epigenomic maps of human tissues, respectively. Similarly, to understand the functional consequences of genetic variation and its impact on complex human diseases, the Genotype-Tissue Expression (GTEx) project was initiated in 2010 (141). The Enhancing GTEx (eGTEx) project was later introduced to complement gene expression phenotypes determined in the GTEx project by extending data depth and introducing new methods (142). The Cancer Genome Atlas, which includes genomic, epigenomic, transcriptomic, proteomic, and clinical data for 32 cancers, is another landmark multi-omics study that has revolutionized precision oncology (143).

**Table 1:** Multi-omics repositories for precision medicine research

Consortium	Year of launch	Status	Sample size	Omics assays	Reference
The *FANTOM* Consortium	2000	Healthy	Variable	CAGE, RNA-Seq, RADICL-Seq	https://fantom.gsc.riken.jp/
ENCODE	2003	Healthy, Cancer	Variable	RNA-Seq, Chip-Seq, DNase-Seq, eCLIP-Seq, ChIA-PET, Hi-C, CAGE, ScRNA-Seq, ATAC-Seq	https://www.encodeproject.org/
Roadmap Epigenomics	2007	Healthy	Variable	RNA-Seq, ChIP-Seq, DNase-Seq, methylation	http://www.roadmapepigenomics.org/
1000 Genomes Project	2007	Healthy	1000	WGS, Targeted Exome sequencing	https://www.internationalgenome.org/
UK Biobank	2007	Various	500,000	Genotyping, WES, WGS	https://www.ukbiobank.ac.uk/
GTEx and eGTEx	2010	Healthy	948	WGS, WES, RNA-Seq,	https://www.gtexportal.org/home/
eQTLGen consortium	2018	Various	31,684	Whole genome, Transcriptomics	https://www.eqtlgen.org/
MoTrPAC	2019	Healthy	Variable	RNA-seq, ATAC-seq, Methyl-cap, RRBS, WGS, Proteomics, Lipidomics and Metabolomics	https://www.motrpac.org/
All of Us	2020	Healthy	1 million	Surveys, wearables, physical measurements, EHR	https://www.researchallofus.org/
COSMIC	2004	Cancer	Variable	Genomics, Epigenomics, Transcriptomics	https://cancer.sanger.ac.uk/cosmic
TCGA	2006	Cancer	20,000	Genomics, Epigenomics, Transcriptomics	https://portal.gdc.cancer.gov/
CPTAC	2011	Cancer	Variable	Copy number variation, whole genome and whole exome sequencing, DNA methylation, RNA-seq, miRNAs, global proteome, phosphoproteome, acetylome and ubiquitinome, and immune subtyping	https://cptac-data-portal.georgetown.edu/cptacPublic/
TARGET	2016	Pediatric cancers	Variable	Clinical, genomic, transcriptomic, and epigenomic data	https://ocg.cancer.gov/programs/target
ADNI	2004	Alzheimer’s disease patients, mild cognitive impairment subjects, and elderly controls	Variable	Clinical, genetic, magnetic resonance imaging, and positron emission tomography imaging	https://adni.loni.usc.edu/
CommonMind	2012	Schizophrenia, bipolar disorder, and unaffected controls	1000	RNA and DNA sequencing, genotyping, epigenetics	https://www.nimhgenetics.org/resources/commonmind
PsychENCODE	2015	Neuropsychiatric disease	Variable	WGS, Transcriptomics	https://psychencode.synapse.org/
AMP-PD	2018	Alzheimer’s disease, type 2 diabetes, rheumatoid arthritis, systemic lupus erythematosus and Parkinson’s disease	Variable	Transcriptomics, epigenomics, whole genome sequencing, metabolomics, and proteomics	https://amp-pd.org/about

ATAC, Assay for Transposase-Accessible Chromatin; CAGE, Cap Analysis of Gene Expression; ChIA-PET, Chromatin Interaction Analysis by Paired-End Tag; Chip, Chromatin immunoprecipitation; eCLIP, enhanced Crosslinking and Immunoprecipitation; EHR, Electronic Health Record; Hi-C, High-throughput chromosome conformation capture; RADICL, RNA and DNA Interacting Complexes Ligated; RRBS, Reduced-Representation Bisulfite sequencing; ScRNA, Single-cell RNA; WGS, Whole Genome Sequencing.

## Advances and Outlook of Computational Methods in Multi-Omics Data Integration

Heterogeneous and high dimensional nature of multi-omics data requires robust integrative approaches to avoid information burden from an individual data type. Several machine learning methods, including unsupervised (matrix factorization, Bayesian, network-based, and kernel-based) and supervised approaches (multi-staged, multidimensional) have been successfully applied for fast and efficient integrative analysis of multi-omics data and are commonly used in research settings as described in the previous sections (Fig. 2) (144, 145, 146). Supervised approaches relay on labeled data (train data) to learn the underlying patterns and discern similar patterns in the independent data set (test data). Supervised approaches include random forests, hidden Markov models, decision trees, support vector machines, elastic nets, and neural networks among others. Supervised approaches are ideal for the prediction of continuous tasks such as survival or pain scores and the classification of discrete outcomes such as disease/healthy status. Unsupervised approaches discern patterns in the data without the need for labeled data and in an unbiased manner. Unsupervised approaches include principal component analysis (PCA), hierarchical clustering, self-organizing maps, and k-means clustering among others. Unsupervised approaches are well suited for the discovery of disease subtypes, biomarkers, and early diagnosis of disease. A comprehensive list of tools for multi-omics data integration can be found at https://github.com/mikelove/awesome-multi-omics. Moving forward, multi-omics data will be increasingly utilized for precision medicine framework, where incorporation of deep learning, artificial intelligence, and cloud-computing systems will play a crucial role in integrative analysis, interpretation, and visualization of multi-omics, imaging, clinical, wearable, and epidemiological data. Additionally, this has the potential to provide clinicians with automated, real-time, and interpretable platforms in assisting disease diagnosis, treatment strategy, and prognosis.

## Challenges and Future Prospects

Individual omics like WGS and WES have already entered clinics for routine genetic screening, understanding response to treatments, and discovering disease biomarkers (2, 3, 4, 5). However, the clinical implementation of multi-omics for precision health has been challenging for several practical reasons. Firstly, omics data acquisition and analysis require specialized equipment, trained personnel, and large financial commitments. Secondly, while cost and turnaround times are rapidly declining, there are parallel challenges in data storage and analysis. Multi-omics data is often heterogenous and poses myriad challenges associated with “Big data,” that is, volume, variety, velocity, and veracity. Datasets with thousands of variables come with the “curse of dimensionality” where the variance between samples becomes large and sparse, rendering clustering analysis uninformative and posing further challenges in interpreting integrated data (147). In addition, missing values, lack of samples, data complexity, class imbalance, dataset shifts, batch effects, and unavailability of some data types can pose significant challenges. In this, there is a lack of standardization for sample collection and omics data analysis. Third, the heterogenous nature of datasets requires rigorous statistical tools to integrate and interpret the results. As data complexity grows with the inclusion of wearables and EHR data, approaches for deep learning, data mining, and artificial intelligence will be necessary to integrate and interpret them. Fourth, multi-modal data complexity and scale (multi-omics data sets can easily exceed tera byte (TB) scale) require robust data management systems to ensure adequate data handling capacity, privacy, and security. Health management platforms like Personal Health Dashboard (PHD), which utilizes state-of-the-art security and scalable technologies to provide an end-to-end solution for big biomedical data analytics both at an individual and cohort level, were developed to meet these challenges. PHD can also be used for collecting and visualizing diverse data types (wearable, clinical, omics) as demonstrated recently in the investigation of insulin resistance and the detection of pre-symptomatic COVID-19 (59, 60, 148). Similar data management infrastructure tools have been proposed for the integration of imaging data and omics data (149, 150). Fifth, so far multi-omics analysis has been typically restricted to few hundreds of participants rising questions on the scalability. To this end, recent studies have demonstrated the promise of expanding it to large populations (>4000) (151). In addition to these limitations, there is also a general resistance to change among health-care systems and policy makers, who need robust evidence for adopting multi-omics widely. In addition, training among clinicians for interpreting multi-omics results is currently lacking, as is training for scientists to flexibly work across different ‘omes.

In addition to the practical considerations listed earlier, there are equally important ethical considerations surrounding discrimination, consent for testing, data privacy, security, data aggregation, and data re-use. As individualized medical big data becomes commonly utilized in health care settings for personalized medicine, clarity on data ownership, management, distribution, and access needs carefully considered. For example, while data leading to diagnoses are of interest to providers and payers, patients have a right to their privacy. Access to personal medical big data revealing debilitating or expensive disease conditions might prompt employers and insurers to discriminate a person. Thus, legal frameworks are needed to protect privacy as well as providing minimum information necessary for other stake holders (152). Additionally, cloud-based data management platforms hosting personal data must have rigorous regulatory compliance (For *e.g.*, HIPAA, GDPR) to prevent inadvertent/malicious access to data. Some of the commercially available cloud-based platforms such as AWS, Google cloud and MS genomics (www.microsoft.com/en-us/genomics/) have approved and necessary tools for multi-omics data management (153).

Despite these hurdles, with an ongoing decrease in omics analysis costs, availability of robust computational tools for data analysis and management, and integration of data informatics in health-care systems, and adequate training of clinicians, it is projected that by 2030, multi-omics–based precision medicine will increasingly transform clinical medicine with the routine use of multi-omics, microbiome analysis, real-time monitoring of environmental exposures, wearable based continuous monitoring of physical activity, sleep, and metabolic parameters for better management of health (154).

## Conflict of interest

M. S. is a cofounder and scientific advisor of Personalis, SensOmics, Qbio, January AI, Fodsel, Filtricine, Protos, RTHM, Iollo, Marble Therapeutics and Mirvie. He is a scientific advisor of Genapsys, Jupiter, Neuvivo, Swaza, Mitrix.
